# Assessment of carnitine excretion and its ratio to plasma free carnitine as a biomarker for primary carnitine deficiency in newborns

**DOI:** 10.1002/jmd2.12334

**Published:** 2022-09-16

**Authors:** Loek L. Crefcoeur, M. Rebecca Heiner‐Fokkema, Rose E. Maase, Gepke Visser, Monique G. M. de Sain‐van der Velden

**Affiliations:** ^1^ Department of Metabolic Diseases, Wilhelmina Children's Hospital University Medical Center Utrecht Utrecht The Netherlands; ^2^ Division of Metabolic Diseases, Amsterdam Gastroenterology and Metabolism, Emma Children's Hospital Amsterdam UMC, Location University of Amsterdam Amsterdam The Netherlands; ^3^ Department of Laboratory Medicine University of Groningen, University Medical Center Groningen Groningen The Netherlands; ^4^ Department Biologicals, Screening and Innovation Dutch National Institute for Public Health and the Environment Bilthoven The Netherlands; ^5^ Section Metabolic Diagnostics, Department of Genetics University Medical Centre Utrecht Utrecht The Netherlands

**Keywords:** carnitine, FCE, newborn screening, PCD, plasma, ratio, urine

## Abstract

In the Netherlands, newborns are referred by the newborn screening (NBS) Program when a low free carnitine (C0) concentration (<5 μmol/l) is detected in their NBS dried blood spot. This leads to ~85% false positive referrals who all need an invasive, expensive and lengthy evaluation. We investigated whether a ratio of urine C0 / plasma C0 (Ratio_U:P_) can improve the follow‐up protocol for primary carnitine deficiency (PCD). A retrospective study was performed in all Dutch metabolic centres, using samples from newborns and mothers referred by NBS due to low C0 concentration. Samples were included when C0 excretion and plasma C0 concentration were sampled on the same day. Ratio_U:P_ was calculated as (urine C0 [μmol/mmol creatinine])/(plasma C0 [μmol/l]). Data were available for 59 patients with genetically confirmed PCD and 68 individuals without PCD. The Ratio_U:P_ in PCD patients was significantly higher (p value < 0.001) than in those without PCD, median [IQR], respectively: 3.4 [1.2–9.5], 0.4 [0.3–0.8], area under the curve (AUC) 0.837. Classified for age (up to 1 month) and without carnitine suppletion (PCD; *N* = 12, Non‐PCD; *N* = 40), medians were 6.20 [4.4–8.8] and 0.37 [0.24–0.56], respectively. The AUC for Ratio_U:P_ was 0.996 with a cut‐off required for 100% sensitivity at 1.7 (yielding one false positive case). Ratio_U:P_ accurately discriminates between positive and false positive newborn referrals for PCD by NBS. Ratio_U:P_ is less effective as a discriminative tool for PCD in adults and for individuals that receive carnitine suppletion.

## 1 INTRODUCTION

Acylcarnitines are valuable metabolites used in newborn screening (NBS), as abnormal plasma acylcarnitine profiles are indicative of several inborn errors of metabolism (IEM).^1^, ^2^ In case of carnitine depletion, theoretically, the measured concentration of other acylcarnitines may also be reduced, which in turn could give rise to false negative reporting.^3^, ^4^ C0 is therefore considered essential for reliable interpretation of the acylcarnitine profile. All newborns with a C0 concentration <5 μmol/l are referred to a paediatric metabolic specialist for evaluation.^5^

In the past 15 years, in the Netherlands ~15% of newborns referred for follow up of low C0 were diagnosed with primary carnitine deficiency (PCD; OMIM no. 212140). In the remaining 85% no underlying cause for the low C0 was identified and they were able to retain normal plasma C0 concentrations without carnitine supplementation. Furthermore, no other IEM was identified in any of these false positive referrals. Follow‐up of these false‐positive referrals is challenging, requiring a long period of confirmatory testing, which may include measurement of residual carnitine transporter activity in cultured fibroblasts and/or sequencing of the *SLC22A5* gene.^6^ The prolonged uncertainty regarding the health of their newborn child during this extensive evaluation, can cause significant anxiety in the families concerned.^7^ A faster exclusion of the diagnosis PCD may reduce harm by false‐positive referral.

PCD is an autosomal recessive disorder caused by variations in the *SLC22A5* gene, encoding the Organic Cation Transporter Novel 2 (OCTN2) protein.^8^, ^9^ OCTN2 maintains intracellular C0 concentrations by transporting carnitine into cells, and reabsorbing C0 in the renal tubuli.^10^, ^11^, ^12^ Defects in OCTN2 cause a decrease of plasma (and intracellular) C0 concentrations and an increased renal waste by inadequate tubular reabsorption.^10^ This causes a shifted balance of plasma C0 versus C0 excretion in PCD patients, which can be determined by calculating the fractional carnitine excretion (FCE) as: ((urine C0 × plasma creatinine[Cr])/(plasma C0 × urine Cr)) × 100%. FCE has been shown to be increased in PCD patients, ranging from 3% to 190%, with proposed normal ranges of <4%. Whilst FCE may be a promising marker for early differentiation of healthy newborns and PCD‐affected newborns, information on FCE is limited, as most case descriptions at the time of writing are derived from conference abstracts, with restricted descriptions of control groups, timing of sampling and circumstances at sampling (e.g., with or without carnitine supplementation).^13^, ^14^, ^15^, ^16^, ^17^

In the Netherlands, plasma Cr is not routinely measured in newborns referred for low C0. However, in most Dutch metabolic specialist centres, plasma C0, C0 excretion and urine Cr are measured upon referral. We investigated whether a ratio of C0 excretion to plasma C0 could be an effective diagnostic tool at the time of referral to improve and simplify the follow‐up protocol for PCD.

## 2 MATERIALS AND METHODS

### 2.1 Study design

Urine and plasma C0 results were retrospectively collected. Inclusion criteria were: (1) C0 excretion and plasma C0 were sampled on the same day and (2) sampled individuals were referred after identification by NBS (newborns and mothers of newborns referred by NBS). The following data were collected: C0 and Cr excretion, plasma C0 concentration, age at sampling, sex, received carnitine suppletion at the time of sampling and PCD diagnosis (confirmed genetically, presented in Table S1). Written informed consent was obtained from all participants and/or their caregivers (METC protocol number 19‐234/M). The study was conducted in accordance with the principles of the Declaration of Helsinki.

### 2.2 Calculation of carnitine excretion in relation to plasma carnitine

The following equation was used to calculate the ratio of urine C0 to plasma C0 (Ratio_U:P_):

$${\text{Ratio}}_{U:P}=\frac{\text{Urine}\;C0\;\left(\text{μmol}/\text{mmol}\;\mathrm{Cr}\right)\;}{\text{Plasma}\;C0\;\left(\text{μmol}/l\right)}$$

### 2.3 Diagnostic accuracy analysis

Receiver operator characteristic (ROC) curve analysis was performed for plasma C0, C0 excretion and Ratio_U:P_, with PCD as the classifier. ROC curve analysis was then repeated in data classified for carnitine suppletion (with or without suppletion at time of sampling) and age (age at sampling up to 1 month and age at sampling above 1 month).

### 2.4 Statistics

All analyses were performed using R (version 4.1.3, R Core Team, Vienna, Austria). DeLong's test was used to compare two ROC curves. The Mann–Whitney *U*‐test was used to compare continuous data between PCD and non‐PCD. The chi‐squared test was used to compare two categorical data between two groups. Significance was assumed for p < 0.05.

## 3 RESULTS

Baseline characteristics are provided in Table 1. In the non‐PCD (*n* = 68) versus the PCD (*n* = 59) population, respectively, the median [interquartile range (IQR)] plasma C0 concentration was 11.1 [8.6–17.7] and 9.0 [6.7–22.0] μmol/l, median C0 excretion was 5.0 [3.0–11.0] and 34.5 [9.6–127.6] μmol/mmol Cr and median Ratio_U:P_ was 0.4 [0.3–0.8] and 3.4 [1.2–9.5]. The non‐PCD population contained more samples from newborns (*N* = 54 [79.4%] vs. PCD: *N* = 27 [45.8%]) and a lower age at sampling (13 days vs. PCD: 9215 days [25 years]). In the non‐PCD population, six (8.8%) received carnitine suppletion at the time of sampling, versus 22 (37.3%) in the PCD population.

**TABLE 1:** Baseline characteristics

	No PCD		PCD		p Value
	*N* = 68		*N* = 59		
	*N*/Median (%)/[IQR]	Range	*N*/Median (%)/[IQR]	Range	
Gender (male, *N*)	19 (27.9)		12 (20.3)		0.431
Referral					<0.001
Newborn (N)	54 (79.4)		27 (45.8)		
Maternal (N)	14 (20.6)		32 (54.2)		
Age at sampling					
Age (median, days)	13 [10–92.3]	7–13 400	9215 [53–12 319]	5–14 600	<0.001
Age <1 month (*N*)	40 (58.8)		15 (25.4)		<0.001
Plasma C0 concentration (median, μmol/l)	11.1 [8.6–17.7]	3.2–79	9.0 [6.7–22.0]	3.6–73.0	0.087
C0 excretion (median, μmol/mmol Cr)	5.0 [3.0–11.0]	0–1460	34.5 [9.6–127.6]	0.7–1520	<0.001
Ratio_U:P_ (median)	0.4 [0.3–0.8]	0–31.6	3.4 [1.2–9.5]	0.06–31.0	<0.001
On carnitine suppletion (yes, *N*)	6 (8.8)		22 (37.3)		<0.001

Abbreviations: C0, free carnitine; Cr, creatinine; PCD, primary carnitine deficiency.

### 3.1 Diagnostic accuracy of Ratio_U_ _:P_

**FIGURE 1:**
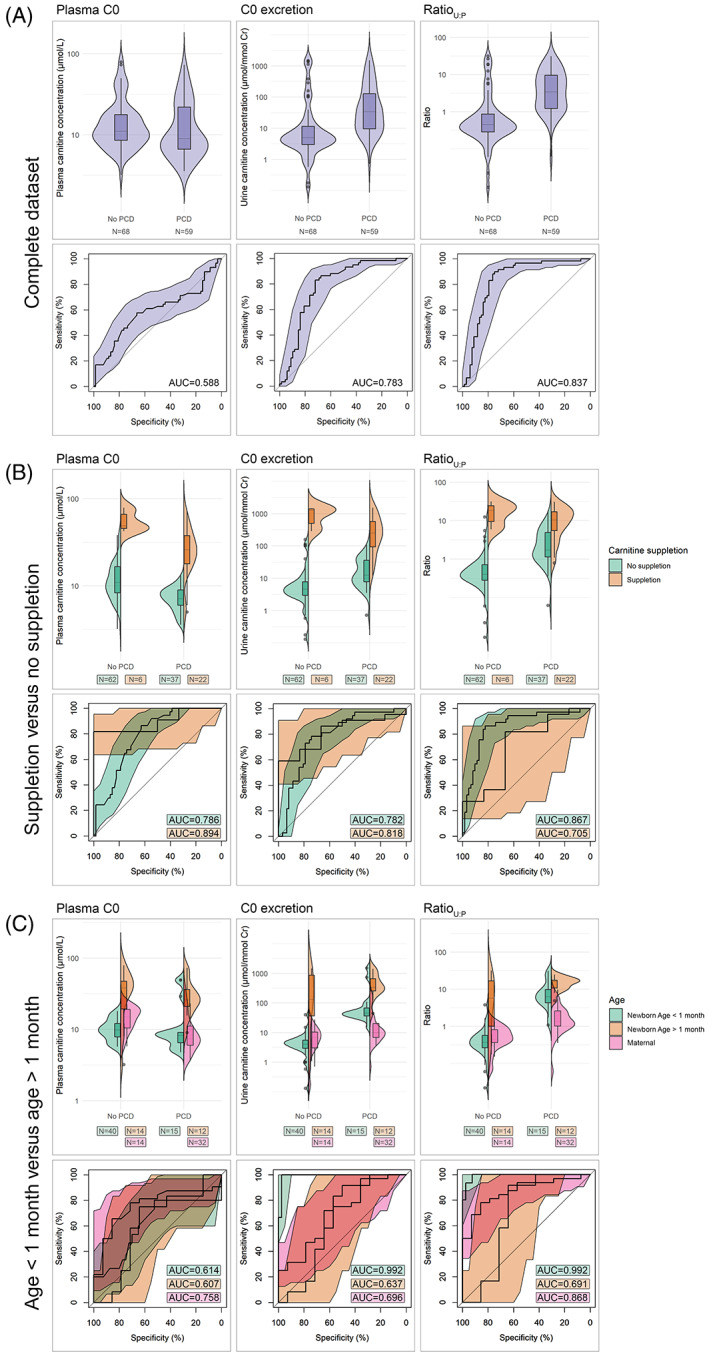
Violin plots and corresponding ROC‐curves (with 95% confidence interval) for plasma C0, C0 excretion and Ratio_U:P_ in (A) the complete dataset, (B) dataset classified for suppletion and (C) dataset classified for age at sampling. AUC, area under the curve; C0, carnitine; Cr, creatinine; PCD, primary carnitine deficiency; ROC, receiver operator characteristic

Unclassified, area under the curve (AUC)'s [95%CI] for plasma C0, C0 excretion and Ratio_U:P_ were: 0.588 [0.485–0.691], 0.783 [0.701–0.866] and 0.837 [0.763–0.912], respectively (Figure 1A). To further evaluate the accuracy of Ratio_U:P_ in the target population (non‐supplemented newborns), data were classified for carnitine suppletion (with or without suppletion; Figure 1B) as well as age (age ≤1 month and age >1 month; Figure 1C). AUC for Ratio_U:P_ increased in an unsupplemented population (0.867 [0.790–0.943] vs. 0.705 [0.437–0.973]) and in a population with an age up to 1 month (0.992 [0.977–1] vs. 0.691 [0.464–0.917] for newborns sampled at age above 1 month and 0.868 [0.763–0.974] for maternal samples).

**FIGURE 2:**
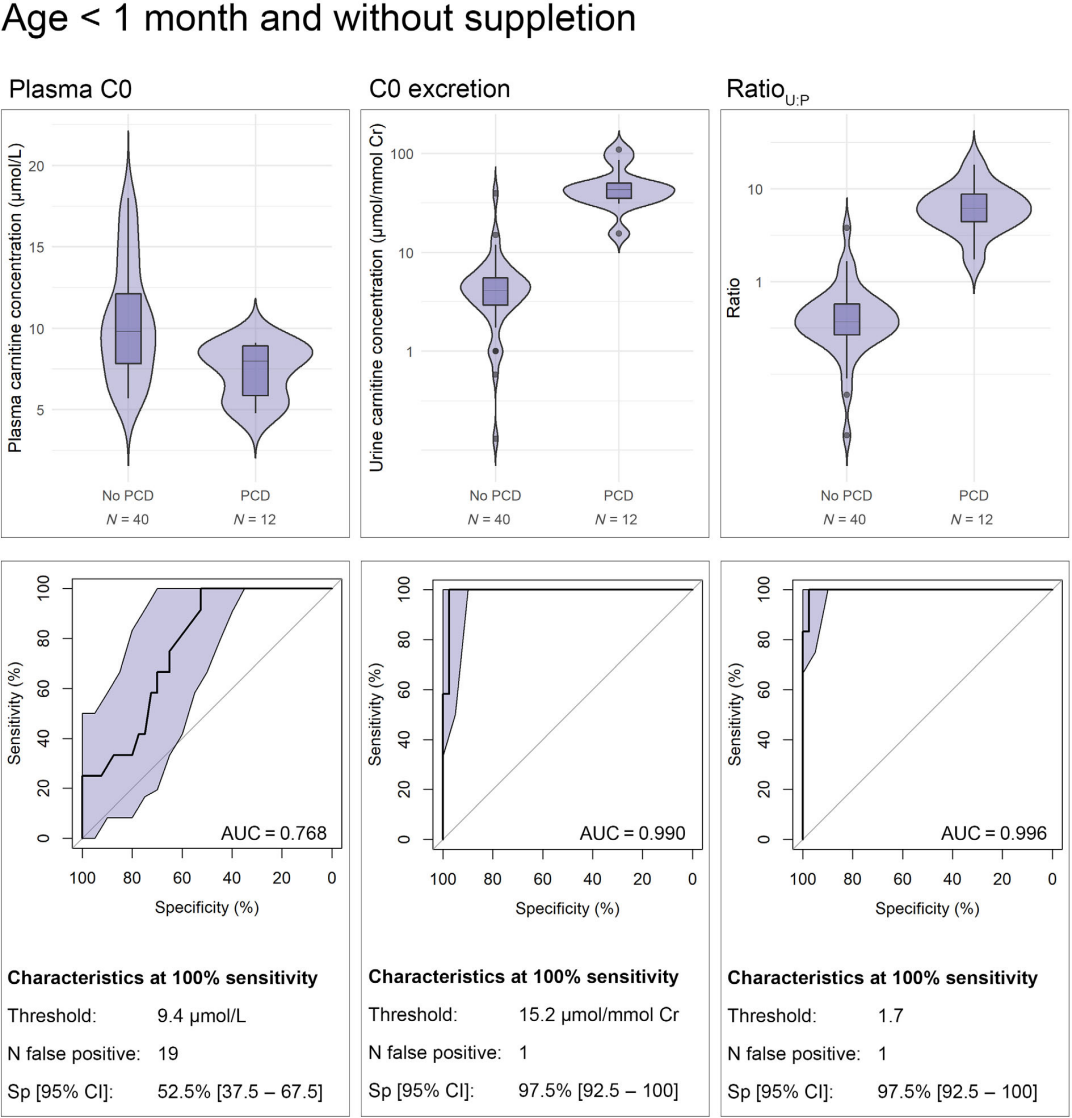
Violin plots and corresponding ROC‐curves (with 95% CI) for plasma C0, C0 excretion and ratio_U:P_ in newborns referred for low C0 in NBS, sampled before age 1 month and without carnitine suppletion at time of sampling. Provided with ROC analysis for each test are the diagnostic characteristics at the threshold for 100% sensitivity. AUC, area under the curve; C0, carnitine; CI, confidence interval; Cr, creatinine; NBS, newborn screening; PCD, primary carnitine deficiency; ROC, receiver operator characteristic; Sp, specificity

The final analysis, in an unsupplemented population with an age up to 1 month (median age at sampling: 11 days), demonstrated the following median [IQR] for non‐PCD (*n* = 40) and PCD (*n* = 12), respectively: plasma C0 9.8 [7.8–12.1] and 8.0 [5.9–8.9] (p = 0.005), C0 excretion 4.0 [2.8–5.4] and 43.0 [35.1–50.2] (p < 0.001), Ratio_U:P_ 0.37 [0.24–0.56] and 6.2 [4.4–8.8] (p < 0.001). The AUC's [95% CI] for plasma C0, C0 excretion and Ratio_U:P_ were respectively: 0.768 [0.634–0.901], 0.990 [0.968–1], 0.996 [0.986–1] (Figure 2). The corresponding thresholds for 100% sensitivity were 9.4 μmol/l, 15.2 μmol/mmol Cr, 1.7, respectively. The specificity [95% CI] at 100% sensitivity for C0 excretion and Ratio_U:P_ both was 92% [92.5%–100%] (yielding one false positive).

## 4 DISCUSSION

This study demonstrates that the ratio of C0 excretion to plasma C0 can be used as an early discriminative test for PCD in newborns referred by NBS. In our study population, applying a threshold of 1.7 the Ratio_U:P_ yields 12 true positive cases, 1 false positive case and correctly identifies as negative 39 newborns that do not have PCD (median ratio in healthy newborns is 0.37). Using the Ratio_U:P_ significantly decreases the duration of follow‐up, cost and harm of false positive referrals by NBS.

PCD is implemented in several NBS programs across the world^1^, ^18^, ^19^, ^20^ and NBS for PCD is known to have a poor positive predictive value (PPV), ranging from 1.6% to 4.7%.^21^, ^22^ In part due to the high false positive rate, the benefit of screening for PCD remains in question.^22^, ^23^ The high false positive rate, in addition to the identification of many asymptomatic individuals, led to discontinuation of screening for PCD in New Zealand.^23^ Detection of PCD following NBS in the Netherlands performs slightly better (PPV 14.6%), which might be due to a different NBS procedure. If C0 concentration is low in the DBS, a second NBS sample is taken and newborns are only referred when the low C0 concentration persists.^5^ However, this algorithm comes at the cost of timeliness. In the Netherlands initial DBS's are obtained 72–168 h after birth and in the event a second sample is requested, there is a further delay of 2–3 days before a newborn is referred.^1^, ^18^, ^19^, ^20^ Ratio_U:P_ does not improve the PPV of NBS for PCD, but it can mitigate the harm of the high amount of false positive referrals by quickly ruling out PCD.

The difference in relative carnitine excretion is less pronounced in supplemented individuals (Figure 1B). This is expected, as the plasma concentration threshold for tubular carnitine reabsorption is 40–60 μmol/l.^24^ Therefore, supplemented healthy individuals could quickly increase their relative carnitine excretion, approaching values of PCD patients (with or without suppletion). Indeed, the median plasma C0 concentration of healthy supplemented individuals was 49 μmol/l (range 42.6–79), with a Ratio_U:P_ of 18.8 (range 5.9–31.6; Table S2). Therefore, we recommend using the Ratio_U:P_ as a test prior to initiating carnitine supplementation.

Ratio_U:P_ is less effective as a discriminative test in a population above the age of 1 month (Figure 1C). It is important to note that many of the newborns in this category received carnitine suppletion when sampled (11/12 PCD and 6/14 non‐PCD), and as discussed above, this may have confounded the results in this population. The other part of this population consists of mothers, sampled ~1–2 weeks after delivery (*N* = 46 out of 72). During the first trimester, glomerular filtration rate increases and slowly recovers 6–8 weeks after delivery.^25^ This may cause an underestimation of Ratio_U:P_ in PCD‐affected mothers. Furthermore, maternal PCD cases identified through NBS likely have milder phenotypes, as many are reported to be completely asymptomatic at the time of diagnosis.^26^, ^27^, ^28^, ^29^ A milder defect may result in less pronounced renal C0 wasting, leading to a lower Ratio_U:P_ (median Ratio_U:P_ maternal PCD: 1.35, newborn PCD: 9.83; Table S3). Last, in general, with increasing age, the population becomes less homogenous, with more diverse renal functions, body masses and diets that may all differently impact carnitine homeostasis.

Thus far, only FCE has been investigated as a diagnostic parameter expressing relative carnitine excretion.^14^, ^15^, ^16^, ^17^ Theoretically, FCE may be superior to Ratio_U:P_, as it corrects for Cr excretion by taking plasma Cr levels into account. However, for the purpose of fast exclusion of PCD, this correction is only beneficial (compared with Ratio_U:P_) when Cr‐excretion is increased due to elevated plasma levels of Cr—in that case, C0 excretion and Ratio_U:P_ would appear low, whereas FCE would still be elevated, due to the high plasma Cr in the numerator of the equation. Causes for primarily elevated plasma Cr include increased muscle degradation (e.g., due to a myopathy) and strenuous exercise, which are extremely rare in newborns and are therefore unlikely to cause false negatives when using Ratio_U:P_.^30^ Other causes for Cr disturbances are renal insufficiency, where plasma Cr rises, as urine Cr decreases,^31^ which would lead to a perceived increase of both FCE and Ratio_U:P_ or glomerular hyperfiltration, where urine Cr is increased and plasma Cr decreases, which would lead to a perceived decrease of both FCE and Ratio_U:P_.^25^, ^32^ Unfortunately, we could not compare FCE to Ratio_U:P_ in our NBS population, as plasma Cr was often not available. Future research comparing FCE to Ratio_U:P_ as a marker for PCD is required.

We acknowledge the limitations of using the ratio_U:P_ as a discriminative tool for PCD. First, carnitine in urine is not routinely available for clinical decision makers and larger studies are necessary to validate the threshold. Finally, immaturity of renal absorption may result in decreased ability of kidneys to preserve carnitine. Interestingly, solely C0 excretion can accurately discriminate PCD from false‐positives in an unsupplemented, newborn population (with an AUC of 0.990). The added benefit of C0 excretion in NBS has been reported previously in a conference abstract by Gallant et al.,^33^ demonstrating the following C0 excretion (nmol/mg Cr) (median [range]): PCD 401 [45–1030], false positives 28 [7–516]. They report increased accuracy when combining newborn plasma and urine C0 concentrations. As C0 excretion and plasma C0 are generally sampled simultaneously we would recommend using the ratio_U:P_ rather than urine excretion values.

In conclusion, the ratio of urinary free carnitine over plasma free carnitine can effectively discriminate between true and false positive referrals from NBS for PCD reducing time to diagnosis and mitigating the negative effects of a false positive referral. The ratio is most effective in neonates under 1 month of age and prior to carnitine supplementation.

## AUTHOR CONTRIBUTIONS

Loek L. Crefcoeur, Monique G. M. de Sain‐van der Velden and M. Rebecca Heiner‐Fokkema collected data. Loek L. Crefcoeur analysed and interpreted the data. Loek L. Crefcoeur and Monique G. M. de Sain‐van der Velden drafted the article. M. Rebecca Heiner‐Fokkema, Rose E. Maase and Gepke Visser provided valuable input for finalising the article.

## CONFLICT OF INTEREST

Loek Crefcoeur, Rebecca Heiner, Rose Maase, Gepke Visser and Monique de Sain declare no potential conflicts of interest.

## ETHICS STATEMENT

All procedures followed were in accordance with the ethical standards of the responsible committee on human experimentation (institutional and national) and with the Helsinki Declaration of 1975, as revised in 2000 (Medical Research Ethics Committee Utrecht; METC protocol number 19‐234/M). Written informed consents of all included individuals were obtained.

## Supporting information


**TABLE S1** Genetic variants of the PCD patients in the study cohort

**TABLE S2** Characteristics of individuals with and without suppletion

**TABLE S3** Characteristics of individuals identified by newborn screening (Newborn) or by newborn screening of their child (Maternal)

**TABLE S4** AUC's and 100% sensitivity cut‐off values for all classified groups

## Data Availability

Supporting data are made available on an online open data repository.
