# Supplementary material for: Assessment of carnitine excretion and its ratio to plasma free carnitine as a biomarker for primary carnitine deficiency in newborns
Source: JIMD Rep. 2022 Sep 16;64(1):57–64. doi: 10.1002/jmd2.12334 (PMC9830017; doi:10.1002/jmd2.12334)
Supplement: Supplementary file 1 — TABLE S1 Genetic variants of the PCD patients in the study cohort [file JMD2-64-57-s003.docx]

| **Case number** | **Case type** | **Coding DNA change allele 1** | **Protein change allele 1** | **Coding DNA change allele 2** | **Protein change allele 2** |
| --- | --- | --- | --- | --- | --- |
| 1* | Maternal | c.136C>T | p.Pro46Ser | c.136C>T | p.Pro46Ser |
| 2 | Maternal | c.1354G>A | p.Glu452Lys | c.-149G>A |  |
| 3 | Maternal | c.136C>T | p.Pro46Ser | c.695C>T | p.Thr232Met |
| 4 | Maternal | c.34G>A | p.Gly12Ser | c.-149G>A |  |
| 5 | Maternal | c.-149G>A |  | c.-149G>A |  |
| 6* | Maternal | c.-149G>A |  | c.-149G>A |  |
| 7 | Maternal | c.34G>A | p.Gly12Ser | c.1340A>C | p.Tyr447Ser |
| 8 | Maternal | c.95A>G | p.Asn32Ser | c.-149G>A |  |
| 9 | Maternal | c.136C>T | p.Pro46Ser | c.-149G>A |  |
| 10 | Maternal | c.136C>T | p.Pro46Ser | c.-149G>A |  |
| 11* | Maternal | c.-149G>A |  | c.-149G>A |  |
| 12* | Maternal | c.640_641delinsTT | p.Ala214Leu | c.-149G>A |  |
| 13 | Maternal | c.718G>A | p.Ala240Thr | c.-149G>A |  |
| 14* | Maternal | c.797C>T | p.Pro266Leu | c.797C>T | p.Pro266Leu |
| 15* | Maternal | c.680G>A | p.Arg227His | c.-149G>A |  |
| 16 | Maternal | c.136C>T | p.Pro46Ser | c.-149G>A |  |
| 17* | Maternal | c.136C>T | p.Pro46Ser | c.-149G>A |  |
| 18* | Maternal | c.-149G>A |  | c.707G>A | p.Cys236Tyr |
| 19 | Maternal | c.136C>T | p.Pro46Ser | c.136C>T | p.Pro46Ser |
| 20 | Newborn | c.597delG | p.Phe200Leufs | c.597delG | p.Phe200Leufs |
| 21* | Newborn | c.136C>T | p.Pro46Ser | c.136C>T | p.Pro46Ser |
| 22* | Newborn | c.136C>T | p.Pro46Ser | c.248G>T | p.Arg83Leu |
| 23* | Newborn | c.136C>T | p.Pro46Ser | c.136C>T | p.Pro46Ser |
| 24* | Newborn | c.136C>T | p.Pro46Ser | c.695C>T | p.Thr232Met |
| 25 | Newborn | c.136C>T | p.Pro46Ser | c.-149G>A |  |
| 26 | Newborn | c.506G>C | p.Arg169Pro | c.1088T>C | p.Leu363Pro |
| 27 | Newborn | c.136C>T | p.Pro46Ser | c.136C>T | p.Pro46Ser |
| 28* | Newborn | c.136C>T | p.Pro46Ser | c.136C>T | p.Pro46Ser |
| 29 | Newborn | c.248G>T | p.Arg83Leu | c.248G>T | p.Arg83Leu |
| 30* | Newborn | c.95A>G | p.Asn32Ser | c.136C>T | p.Pro46Ser |
| 31 | Newborn | c.448T>C | p.Phe150Leu | c.760C>T | p.Arg254Ter |
| 32* | Newborn | c.844C>T | p.Arg282Ter | c.-149G>A |  |
| 33 | Newborn | c.248G>T | p.Arg83Leu | c.248G>T | p.Arg83Leu |
| 34* | Newborn | c.610G>A | p.Gly204Ser | c.-149G>A |  |
| 35 | Newborn | c.760C>T | p.Arg254Ter | c.1354G>A | p.Glu452Lys |
| Variants were detected in the *SLC22A5* gene using Sanger sequencing.  * From these patients multiple samples were used in the study; before and after suppletion. | | | | | |

**Supplementary Table 1.** Genetic variants of the PCD patients in the study cohort.
