# Supplementary material for: Assessment of carnitine excretion and its ratio to plasma free carnitine as a biomarker for primary carnitine deficiency in newborns
Source: JIMD Rep. 2022 Sep 16;64(1):57–64. doi: 10.1002/jmd2.12334 (PMC9830017; doi:10.1002/jmd2.12334)
Supplement: Supplementary file 2 — TABLE S2 Characteristics of individuals with and without suppletion [file JMD2-64-57-s001.docx]

**Supplementary Table 2. Characteristics of individuals with and without suppletion.**

|  | **No suppletion** | | | | **Suppletion** | | | |
| --- | --- | --- | --- | --- | --- | --- | --- | --- |
|  | **No PCD** | | **PCD** | | **No PCD** | | **PCD** | |
|  | **(N=62)** | | **(N=37)** | | **(N=6)** | | **(N=22)** | |
| **Sex** (male, N) | 45 | (72.6) | 30 | (81.1) | 4 | (66.7) | 17 | (77.3) |
| **Age at sampling** |  |  |  |  |  |  |  |  |
| **Age** (median, days) | 12.0 | [7.00 - 13400] | 10300 | [5.00 - 14600] | 58.5 | [36.0 - 3040] | 1650 | [18.0 - 13400] |
| **Age < 1 month** (N) | 40 | (64.5) | 12 | (32,4) | 0 | (0) | 3 | (13.6) |
| **Plasma C0 concentration** (median, µmol/L) | 10.9 | [3.20 - 38.4] | 7.11 | [3.58 - 13.0] | 49.0 | [42.6 - 79.0] | 26.1 | [5.00 - 73.0] |
| **C0 excretion** (median, µmol/mmol Cr) | 4.50 | [0 - 159] | 12.6 | [0.721 - 109] | 1260 | [285 - 1460] | 253 | [12.1 - 1520] |
| **Ratio_U:P_** (median) | 0.4 | [0 - 12.5] | 1.74 | [0.06 - 18.2] | 18.8 | [5.9 - 31.6] | 10.4 | [0.8 - 31.0] |
| Values presented as: median [range] or N (%). Abbreviations: PCD - primary carnitine deficiency; C0 - free carnitine; Cr - creatinine | | | | | | | | |
