# Supplementary material for: Assessment of carnitine excretion and its ratio to plasma free carnitine as a biomarker for primary carnitine deficiency in newborns
Source: JIMD Rep. 2022 Sep 16;64(1):57–64. doi: 10.1002/jmd2.12334 (PMC9830017; doi:10.1002/jmd2.12334)
Supplement: Supplementary file 3 — TABLE S3 Characteristics of individuals identified by newborn screening (Newborn) or by newborn screening of their child (Maternal) [file JMD2-64-57-s004.docx]

**Supplementary Table 3. Characteristics of individuals identified by newborn screening (Newborn) or by newborn screening of their child (Maternal)**

|  | **Newborn** | | | | **Maternal** | | | |
| --- | --- | --- | --- | --- | --- | --- | --- | --- |
|  | **No PCD** | | **PCD** | | **No PCD** | | **PCD** | |
|  | **(N=54)** | | **(N=27)** | | **(N=14)** | | **(N=32)** | |
| **Plasma C0 concentration** (median, µmol/L) | 10.9 | [3.20 - 79.0] | 15.6 | [4.77 - 73.0] | 15.2 | [5.77 - 22.2] | 7.39 | [3.58 - 39.0] |
| **C0 excretion** (median, µmol/mmol Cr) | 4.58 | [0 - 1460] | 109 | [15.4 - 1520] | 6.79 | [0.176 - 27.6] | 10.0 | [0.721 - 237] |
| **Ratio_U:P_** (median) | 0.430 | [0 - 31.6] | 9.83 | [1.08 - 31.0] | 0.445 | [0.00921 - 1.37] | 1.35 | [0.0619 - 9.81] |
| **Age at sampling** |  |  |  |  |  |  |  |  |
| **Age** (median, days) | 12.0 | [7.00 - 3040] | 18.0 | [5.00 - 3680] | 12000 | [9510 - 13400] | 12100 | [9060 - 14600] |
| **Age < 1 month** (N) | 40 | (74.1) | 15 | (55.6) | 0 | (0) | 0 | (0) |
| **Age > 1 month** (N) | 14 | (25.9) | 12 | (44.4) | 14 | (100) | 32 | (100) |
| **On carnitine suppletion** (yes, N) | 6 | (11.1) | 14 | (51.9) | 0 | (0) | 8 | (25.0) |
| Values presented as: median [range] or N (%). Abbreviations: PCD - primary carnitine deficiency; C0 - free carnitine; Cr - creatinine | | | | | | | | |
