# Supplementary material for: Assessment of carnitine excretion and its ratio to plasma free carnitine as a biomarker for primary carnitine deficiency in newborns
Source: JIMD Rep. 2022 Sep 16;64(1):57–64. doi: 10.1002/jmd2.12334 (PMC9830017; doi:10.1002/jmd2.12334)
Supplement: Supplementary file 4 — TABLE S4 AUC's and 100% sensitivity cut‐off values for all classified groups [file JMD2-64-57-s002.docx]

**Supplementary Table 4.** AUC’s and 100% sensitivity cut-off values for all classified groups

|  |  |  |  |  |  |  |
| --- | --- | --- | --- | --- | --- | --- |
| **Unclassified** | **PCD (N)** | **No PCD (N)** | **Newborn (N)** | **Maternal (N)** |  |  |
|  | 59 | 68 | 81 | 46 |  |  |
|  |  |  |  |  |  |  |
|  | **AUC** | **(95% CI)** |  | **Threshold 100% sensitivity** | **N false positive at 100% sensitivity** |  |
| **Plasma C0** | 0,588 | 0,485 | 0,691 | 73,04 | 66 | 97% |
| **C0 excretion** | 0,783 | 0,701 | 0,866 | 0,71 | 62 | 91% |
| **Ratio_U:P_** | 0,837 | 0,763 | 0,912 | 0,06 | 63 | 93% |
|  |  |  |  |  |  |  |
| **No suppletion** | **PCD (N)** | **No PCD (N)** | **Newborn (N)** | **Maternal (N)** |  |  |
|  | 37 | 62 | 61 | 38 |  |  |
|  |  |  |  |  |  |  |
|  | **AUC** | **(95% CI)** |  | **Threshold 100% sensitivity** | **N false positive at 100% sensitivity** |  |
| **Plasma C0** | 0,786 | 0,698 | 0,874 | 13,07 | 38 | 61% |
| **C0 excretion** | 0,782 | 0,688 | 0,875 | 0,71 | 56 | 90% |
| **Ratio_U:P_** | 0,867 | 0,790 | 0,943 | 0,06 | 57 | 92% |
|  |  |  |  |  |  |  |
| **Suppletion** | **PCD (N)** | **No PCD (N)** | **Newborn (N)** | **Maternal (N)** |  |  |
|  | 22 | 6 | 20 | 8 |  |  |
|  |  |  |  |  |  |  |
|  | **AUC** | **(95% CI)** |  | **Threshold 100% sensitivity** | **N false positive at 100% sensitivity** |  |
| **Plasma C0** | 0,894 | 0,773 | 1,000 | 73,0 | 4 | 67% |
| **C0 excretion** | 0,818 | 0,651 | 0,986 | Inf | 6 | 100% |
| **Ratio_U:P_** | 0,705 | 0,437 | 0,973 | 31,3 | 5 | 83% |
|  |  |  |  |  |  |  |
| **Age < 1 month** | **PCD (N)** | **No PCD (N)** | **Newborn (N)** | **Maternal (N)** |  |  |
|  | 15 | 40 | 55 | 0 |  |  |
|  |  |  |  |  |  |  |
|  | **AUC** | **(95% CI)** |  | **Threshold 100% sensitivity** | **N false positive at 100% sensitivity** |  |
| **Plasma C0** | 0,614 | 0,421 | 0,807 | Inf | 40 | 100% |
| **C0 excretion** | 0,992 | 0,974 | 1,000 | 15,22 | 1 | 3% |
| **Ratio_U:P_** | 0,992 | 0,977 | 1,000 | 1,07 | 3 | 8% |
|  |  |  |  |  |  |  |
| **Age > 1 month** | **PCD (N)** | **No PCD (N)** | **Newborn (N)** | **Maternal (N)** |  |  |
|  | 44 | 28 | 26 | 46 |  |  |
|  |  |  |  |  |  |  |
|  | **AUC** | **(95% CI)** |  | **Threshold 100% sensitivity** | **N false positive at 100% sensitivity** |  |
| **Plasma C0** | 0,689 | 0,563 | 0,815 | 73,04 | 24 | 86% |
| **C0 excretion** | 0,567 | 0,419 | 0,714 | 0,71 | 24 | 86% |
| **Ratio_U:P_** | 0,662 | 0,515 | 0,808 | 0,04 | 25 | 89% |
|  |  |  |  |  |  |  |
| **Final** | **PCD (N)** | **No PCD (N)** | **Newborn (N)** | **Maternal (N)** |  |  |
|  | 12 | 40 | 52 | 0 |  |  |
|  |  |  |  |  |  |  |
|  | **AUC** | **(95% CI)** |  | **Threshold 100% sensitivity** | **N false positive at 100% sensitivity** |  |
| **Plasma C0** | 0,768 | 0,634 | 0,901 | 9,37 | 19 | 48% |
| **C0 excretion** | 0,990 | 0,968 | 1,000 | 15,22 | 1 | 3% |
| **Ratio_U:P_** | 0,996 | 0,986 | 1,000 | 1,70 | 1 | 3% |
| Abbreviations: C0 – free carnitine; Ratio_U:P_ – Ratio urine plasma C0 (urine C0 (µmol/mmol creatinine)/ plasma C0 (µmol/L)); PCD – primary carnitine deficiency; AUC – area under the curve. | | | | | | |
